# An Assessment of the Presence of *Clostridium tetani* in the Soil and on Other Surfaces

**DOI:** 10.5811/westjem.18702

**Published:** 2024-10-22

**Authors:** Michael Shalaby, Alessandro Catenazzi, Melissa F. Smith, Robert A. Farrow II, David Farcy, Oren Mechanic, Tony Zitek

**Affiliations:** * Herbert Wertheim College of Medicine at Florida International University, Department of Emergency Medicine and Critical Care, Miami, Florida; † Mount Sinai Medical Center Miami Beach, Department of Emergency Medicine, Miami Beach, Florida; ‡ Catenazzi Lab at Florida International University, Department of Biological Sciences, Miami, Florida

## Abstract

**Introduction:**

Standard emergency medicine practice includes tetanus vaccine administration as part of wound care management for patients who are not fully immunized. Since there have been no available studies in the United States reaffirming the prevalence of *Clostridium tetani (C tetani)* since 1926, we sought to identify its prevalence in a major urban county in the US.

**Methods:**

We sampled soil, rusted metal, concrete, and dog feces to determine the prevalence of *C tetani* in a single metropolitan county in the United States. Soil samples and swabs were collected from four locations: the soil of a public park and an elementary school; dog feces from a single public dog park; and rusted surfaces (metal and concrete) in common student areas of a university campus. The presence of *C tetani* in each sample was determined using a quantitative polymerase chain reaction.

**Results:**

In total, 200 samples were collected, of which 37 (18.5%) tested positive for *C tetani* DNA. Among the 140 samples taken from the soil, just one (0.7%) tested positive for *C tetani* DNA. Of the 40 samples of rusted metal and concrete surfaces, 30 (75%) tested positive for *C tetani*, and six (30%) of the 20 samples from dog feces tested positive for *C tetani*.

**Conclusion:**

We found that *C tetani* is frequently present on rusted metal and concrete surfaces but rarely in soil samples. Minor wounds contaminated with soil may be considered low risk for tetanus. However, future studies should assess the burden of *C tetani* in other similar urban, suburban, and rural environments to help determine the threat of *C tetani* more exactly.

Population Health Research Capsule What do we already know about this issue?

*Clostridium tetani is assumed to be widely present within the soil. The last soil sample study performed in the United States was in 1926, which only showed one positive sample of 62 collected.*

What was the research question?

*What is the prevalence of C. tetani in the soil, in dog feces, and on concrete and metal surfaces within a single urban county?*

What was the major finding of the study?

*Among the 140 samples taken from the soil, just 1 (0.7%) tested positive for C. tetani DNA. Of the 40 samples of rusted metal and concrete surfaces, 30 (75%) tested positive, as did 6 (30%) of 20 samples from dog feces.*

How does this improve population health?

*Certain wound types (ie, soil contamination) may carry a lower risk for C tetani, and the elevated cost of tetanus toxoid administration in the emergency department may be forgone for outpatient vaccination which is much cheaper.*

## INTRODUCTION

### Background

*Clostridium tetani* (*C tetani*), a Gram-positive obligate anaerobe, is the causative agent of tetanus, a disorder that induces uncontrollable muscle spasms (known as tetany) and carries high mortality.^1^ It is prevented by a commonly administered toxoid vaccine.^1^ *C tetani* is thought to inhabit soil, most often in the spore form, through which it can withstand extreme temperatures and volatile environments.^2^ After inoculation of contaminated wounds, the spores proliferate and spawn vegetative bacteria, which release toxins that precipitate the disease’s characteristic symptoms of tetany.^1^

Tetanus poses a considerable risk in developing countries with little access to vaccination. In 2015 there were nearly 57,000 cases of tetanus reported worldwide, with 79% originating in South Asia and sub-Saharan Africa.^3^ The annual incidence of tetanus in the United States, in contrast, is very low. Since the introduction of the tetanus toxoid vaccine in the 1930s, the rate of infection has steadily declined from a peak of 500 in 1950 to no more than 30 cases yearly.^4^ Documented cases are typically in injection drug users and the elderly, who have a higher risk of insufficient antibody titers despite updated vaccination status.^1^ ^,^ ^4^

The US Centers for Disease Control and Prevention Advisory Committee on Immunization Practices (ACIP) recommends tetanus vaccination as part of “routine wound care management” for patients who are not up to date with their vaccination after sustaining a wound.^5^ Protocols for wound characteristics (ie, abrasion vs laceration) are not specified by the ACIP.^6^ However, the American College of Emergency Physicians guidelines differentiate between “minor wounds and superficial burns” and “other wounds”: minor wounds require a booster within 10 years, while “other wounds” require a booster within five years.^7^ Thus, tetanus toxoid is administered liberally in US emergency departments (ED) as part of routine wound care, whether for simple abrasions or more complex wounds.

The prevalence of *C tetani* in the soil has not been measured in the US since 1926, when Damon et al fed cultured soil specimens obtained mostly from farmland to pigs and subsequently monitored them for signs of disease.^8^ More recently in 2008, Bukar et al sampled soil in Nigeria, and via incubation of specimens they demonstrated a 60% incidence of *C tetani*.^9^ However, these studies may not be generalizable to modern US populations. Newer, more robust methods for determining the presence of *C tetani* exist today; furthermore, 83% of people in the US today reside in urban environments^10^ where the burden of *C tetani* in the environment may differ. Thus, the true prevalence of *C tetani* in the modern, urban US environment has yet to be elucidated.

We sought to determine the frequency with which *C tetani* is present in the soil as compared to concrete, metal, and dog feces in a single, major urban county in the US.

## MATERIALS AND METHODS

We assessed environmental samples for the presence of *C tetani* DNA in Miami-Dade County, Florida, which has a population of approximately 2.7 million people.^11^ This study did not include human subjects and thus was exempt from review by the institutional review board. This research received no outside funding.

Eighty soil samples were collected in sterilized Whirl-Pak bags (Filtration Group Corp, Madison, WI) from an urban public park and an elementary school. These sites were chosen based on their distance from each other and their likelihood to represent isolated soil flora within the same county, but not within close enough proximity to be expected to share similar flora. Each soil-sample bag contained three separate samples from within a few inches of soil using three separate plastic spoons that were disposed of after each use. We collected samples this way such that each bag was large enough and that one individual spoon might not “contaminate” the other two samples within the same bag. The other samples, collected by DRYSWAB brush (Medical Wire & Equipment Ltd, Corsham, UK), included 20 samples of dog feces from one dog park, and 60 combined samples of concrete and rusted metal surfaces (such as metal signs and railings, and concrete walkways) at a single public university. A subsequent set of 60 soil samples, also collected by dry swab, were again taken from the same locations as the original soil samples in the sterilized bags. Samples were immediately taken to the processing laboratory after collection.

Samples were analyzed in a university microbiology lab using quantitative polymerase chain reaction (qPCR) following a standardized previously described method (Akbulut et al).^12^ This assay amplifies a 160-base pair fragment of the teNT gene (tetanus toxin) of *C tetani* (GenBank Accession Number X06214, X04436). The tip of each swab was removed and placed into a 1.5 milliliter Eppendorf tube containing 50 microliters (μl) PrepMan Ultra Sample Preparation Reagent (Thermo Fisher Scientific, Waltham, MA). The tube was then incubated for 10 minutes at 100°C in a dry bath, after which it was centrifuged at 13,000 revolutions per minute for three minutes. The initial 80 soil samples were analyzed with 500 milligrams of mixed soil, while the dry swabs were mixed with a reagent in the absence of significant amounts of soil in the sample.

Five μl of DNA was then extracted from the tube and transferred to a 0.6-milliliter (mL) qPCR tube containing 45 μl of 1/10 TE buffer. After mixing, 5 μl of this diluted DNA product was added to a new qPCR tube along with 20 μl of a 62.5:35.5:1:1 mixture of SYBR Green master mix (Thermo Fisher Scientific, Waltham, MA): purified water: forward primer TeNT-F (CCTAGTTTCAAAACTTATTGGCTTATGTAA): reverse primer TeNT-R (CATAATTCTCCTCCTAAATCTGTTAATGAT). The qPCR was performed on a QuantStudioTM 3 Real-Time PCR Instrument (96-well 0.1 mL Block) (Applied Biosystems Inc, Foster City, CA) as follows: two minutes at 50°C, followed by two minutes at 90°C, followed by 51 cycles of 15 seconds at 95°C/1 minute at 56°C, followed by a final 15 seconds at 95°C. Our qPCR was specified for the first 160 base pair fragments of the teNT gene of *C tetani*.

The plate included three distilled water negative controls, one PrepMan negative control, and three serial dilutions of double-stranded, synthetic DNA (gBlock, Integrated DNA Technologies Inc, Coralville, IA) of the teNT gene of *C tetani* (GenBank Accession Number X06214, X04436). Results were analyzed in QuantStudio Design and Analysis Software v1.5.1 (Applied Biosystems Inc, Foster City, CA). No power calculation was performed. With no external funding, the investigators determined we had funds for three plates. The maximum number of samples per plate was 82. We analyzed 200 total samples from the environment to assess for the presence of *C tetani* DNA. In the laboratory setting, the assay we used has nearly 100% sensitivity and specificity for *C tetani* DNA, but it is possible that in environmental samples the presence of additional substances may interfere with it. Therefore, we tagged 42 soil samples with *C tetani* to assess the accuracy of our assay (the control group). We calculated the number (%) of samples that were positive for *C tetani* overall, for each type of environmental sample, and for the control group. We compared the three groups (soil, concrete and metal, and dog feces) in a pairwise fashion with regard to the percentage of samples with *C tetani* DNA using Fisher exact tests.

## RESULTS

Overall, of the 200 samples collected for analysis, 37 (18.5%) tested positive for *C tetani* DNA (Table). The first 80 samples consisted of soil collection in plastic bags from public parks. These 80 samples were all negative for *C tetani* DNA by our analysis. However, given the possibility of interference of soil humic acid with qPCR analysis,^13^ another 60 soil samples were obtained via dry swabs from the same locations (urban park and elementary school) as the original 80. Repeat analysis of the 60 dry swabs of the soil revealed one positive for *C tetani* DNA. Therefore, among the 140 analyzed soil samples (with two different methods of collection), one (0.7%) was found to have *C tetani* DNA. To assess the sensitivity of our assay, we tagged 22 samples of soil in sterilized plastic bags with *C tetani* DNA, and 16 were identified as positives (72.7%). We tagged 20 dry swabs of soil with *C tetani* DNA, and 11 (55%) were positive.

**Table.** Swabs and soil sample results for presence of *Clostridium tetani*.

Sample location and type	Positive samples
All soil: • Park samples • Elementary school samples • Park swabs • Elementary school swabs	1/140 (0.7%) • 0/20 (0%) • 0/60 (0%) • 1/15 (1.7%) • 0/45 (0%)
Dog park feces swabs	6/20 (30%)
Undergraduate campus, oxidized metal and concrete swabs	30/40 (75%)

We collected 40 swabs of combined public oxidized concrete and metal surfaces from a university campus, as well as 20 swabs of dog feces at a single public dog park. Of these, six (30%) of 20 dog feces samples were positive for *C tetani* DNA, and 30 (75%) of 40 swabs of oxidized concrete and metal were positive. A control was created and evaluated by tagging 20 of the metal and concrete samples with *C tetani* DNA. Of the 20 control samples, 15 were positive (75%).

There was a statistically significant difference in the percentage of samples with *C tetani* DNA from the concrete and metal group (75%) as compared to the soil group (0.7%), *P* < 0.001. Additionally, there was a statistically significant difference in the percentage of samples with *C tetani* DNA from the dog feces group (30%) as compared to the soil group (0.7%), *P* < 0.001. Finally, there was a statistically significant difference in the percentage of samples with *C tetani* DNA in the concrete and metal group (75%) as compared to the dog feces groups (30%), *P* < 0.001.

## DISCUSSION

We undertook this study to help ascertain the frequency with which *C tetani* is found in the soil and on other substances in the environment in an urban area in the US. Our results suggest that *C tetani* is much more common on oxidized metal and concrete, as well as dog feces than it is in soil. Our results are consistent with the last assessment of US soil from 1926, which did not strongly suggest that *C tetani* was present. With the paucity of *C tetani* isolated in this sample of US soil from a single county, it is suggested that further investigation into the prevalence of the bacterium is needed. There are broader implications to identifying *C tetani*. For one, this study found that non-soil media may provide a more favorable growth environment for *C tetani*, and soil itself may not be an abundant source. Education efforts for exposure may need to be concentrated on injuries due to rusted metal, concrete, dog feces, and potentially other sources of *C tetani*. Given that our study sample was small, we do not believe that this data alone merits a change in ED vaccination guidelines, especially since the tetanus toxoid vaccine also provides immunization against diphtheria and pertussis, both of which are also significant public health threats. Rather, more data from similar studies is required.

## LIMITATIONS

There are some limitations to this small single-US county study. Firstly, *C tetani* DNA samples were tested via qPCR analysis, rather than incubated, and reagents, such as humic acid, within the soil may have interfered with PCR analysis.^13^ Repeat analysis of dry swabs aimed to mitigate such error, and similar results were produced. Furthermore, the prevalence of *C tetani* should be studied in other urban areas before public health conclusions are made. Farmland and non-urban areas were not studied and, therefore, this cannot be generalized. Oxidized metal and concrete surfaces were analyzed together, and thus the extent to which *C tetani* is present on either surface was not fully assessed in this study. Lastly, while this study contains more data samples than previous similar undertakings, 200 soil samples from only a few separate sites in a single, urban county likely do not fully represent the true extent of the presence of *C tetani* in other environments, such as other sites from within the same urban county and other urban, suburban, and rural environments.

## CONCLUSION

Tetanus poses a significant public health threat. Yet its presence in the soil may not be as significant as is currently assumed, at least not in urban areas, as our findings suggest. In our study, we tested soil, concrete, metal, and dog feces for *C tetani* in a single urban county. The results suggest that *C tetani* is more abundant in oxidized metal and concrete, as well as in dog feces than it is in soil. However, several questions about the prevalence and virulence of *C tetani* remain. Further studies should elucidate the prevalence of *C tetani* in other urban, suburban, and rural sites.

## References

[1] Yen LM Thwaites CL . Tetanus. Lancet (London, England). 2019;393(10181):1657–68. 30935736 10.1016/S0140-6736(18)33131-3

[2] Campbell JI Lam TMY Huynh TL et al . Microbiologic characterization and antimicrobial susceptibility of *Clostridium tetani* isolated from wounds of patients with clinically diagnosed tetanus. Am J Trop Med Hyg. 2009;80(5):827–31. 19407132

[3] Kyu HH Mumford JE Stanaway JD et al . Mortality from tetanus between 1990 and 2015: findings from the global burden of disease study 2015. BMC Public Health. 2017;17(1):179. 28178973 10.1186/s12889-017-4111-4 PMC5299674

[4] Centers for Disease Control and Prevention . About tetanus disease (lockjaw). 2022. Available at: https://www.cdc.gov/tetanus/about/index.html. Retrieved April 27, 2023.

[5] Liang JL Tiwari T Moro P et al . Prevention of pertussis, tetanus, and diphtheria with vaccines in the United States: recommendations of the advisory committee on immunization practices (ACIP). MMWR Recomm Rep. 2018;67(2):1–44. 10.15585/mmwr.rr6702a1 PMC5919600 29702631

[6] Havers FP Moro PL Hunter P et al . Use of tetanus toxoid, reduced diphtheria toxoid, and acellular pertussis vaccines: updated recommendations of the Advisory Committee on Immunization Practices — United States, 2019. Morb Mortal Wkly Rep. 2020;69(3):77–83. 10.15585/mmwr.mm6903a5 PMC7367039 31971933

[7] Tetanus . Available at: https://www.acep.org/siteassets/new-pdfs/policy-statements/immunization-of-adults-and-children-in-the-emergency-department.pdf. 2020. Accessed: June 12, 2023.

[8] Damon SR Payabal LB . Distribution of the spores of bacillus botulinus and bacillus tetani in the soil: 1. In Maryland, J Infect Dis. 1926;39(6):491–501.

[9] Bukar A Mukhtar MD Adam SA . Current trend in antimicrobial susceptibility pattern of *Clostridium tetani* isolated from soil samples in Kano. Bayero J Pure Appl Sci. 2008;1(1):112–5.

[10] Center for Sustainable Systems . U. S. Cities factsheet. Available at: https://css.umich.edu/publications/factsheets/built-environment/us-cities-factsheet. Accessed: April 27, 2023.

[11] Census Reporter . Miami-Dade County, FL. Available at: http://censusreporter.org/profiles/05000US12086-miami-dade-county-fl/. Accessed: April 27, 2023.

[12] Akbulut D Dennis J Gent M et al . Wound botulism in injectors can of drugs: upsurge in cases in England during 2004. Euro Surveill. 2005;10(9):172–4. 16280612

[13] Matheson CD Gurney C Esau N et al . Assessing PCR inhibition from humic substances. Open Enzym Inhib J. 2010;3(1):38–45.

